# Selective Serotonin Reuptake Inhibitors for the Prevention of Post-Stroke Depression: A Systematic Review and Meta-Analysis

**DOI:** 10.3390/jcm10245912

**Published:** 2021-12-16

**Authors:** Daniel Richter, Jeyanthan Charles James, Andreas Ebert, Aristeidis H. Katsanos, Lisa Mazul-Wach, Quirin Ruland, Ralf Gold, Georg Juckel, Christos Krogias

**Affiliations:** 1 Department of Neurology, Ruhr-University Bochum, St. Josef-Hospital, 44791 Bochum, Germany; Jeyanthan.CharlesJames@ruhr-uni-bochum.de (J.C.J.); lisa.mazul@rub.de (L.M.-W.); q.ruland@gmail.com (Q.R.); ralf.gold@rub.de (R.G.); christos.krogias@rub.de (C.K.); 2 Department of Psychiatry, Ruhr-University Bochum, LWL-Klinik, 44791 Bochum, Germany; andreas.ebert@lwl.org (A.E.); g.juckel@lwl.org (G.J.); 3 Division of Neurology, McMaster University and Population Health Research Institute, Hamilton, ON L8S 3L8, Canada; ar.katsanos@gmail.com; 4 Medical Faculty, Ruhr-University Bochum, 44801 Bochum, Germany

**Keywords:** serotonin reuptake inhibitor, stroke, post-stroke depression

## Abstract

There are controversial data on the efficacy and safety profile of selective serotonin reuptake inhibitors (SSRIs) to prevent post-stroke depression (PSD). We performed a systematic search in MEDLINE and SCOPUS databases to identify randomized-controlled trials questioning the use of early SSRI therapy in the post-stroke population and its effect on PSD incidence. We included 6 studies with 6560 participants. We extracted the data on PSD occurrence in association with the treatment arm (SSRI versus placebo), as reported by each study. For safety analysis, we extracted the information on adverse events. A random-effects model was used to calculate the pooled relative risk estimates. Early SSRI therapy was associated with a significant reduction of PSD occurrence compared to placebo (10.4% versus 13.8%; relative risk: 0.75 [95% CI, 0.66–0.86]; absolute risk reduction: 3.4%). SSRI therapy increases the risk of bone fracture (RR 2.28 [95% CI, 1.58–3.30]) and nausea (RR 2.05 [95% CI, 1.10–3.82]) in the post-stroke population. Considering the risk-benefit ratio of early SSRI therapy in the post-stroke population, future research should identify high-risk patients for PSD to improve the risk-benefit consideration of this therapy for use in clinical practice.

## 1. Introduction

Stroke is the prime cause of disability and need for care worldwide [1]. For the individual patient, the consequences are often severe and affect both motor and non-motor aspects. In addition to the functional outcome, the neuropsychiatric effects such as cognitive and affective disorders are decisive for the further biography of stroke patients. In this context, post-stroke depression (PSD) is the most common complication, contributing substantially to the impairment of the quality of life and ability to work after stroke [2,3].

In general, the increase of serotonin in the synaptic gap represents the essential rationale for using serotonin reuptake inhibitors (SSRI) in patients with depression. The largest existing randomized controlled trial (RCT) primary analyzing the use of SSRIs (escitalopram) for the prevention of a PSD found no significant difference between SSRI and placebos [4]. On the other hand, the Cochrane review by Mead suggests an advantage of SSRIs for preventing PSD [5]. However, side effects such as seizures, nausea, and bone fracture seem to occur more frequently under SSRI therapy in the post-stroke population [6,7]. Besides controversial data on the risk of suicide under SSRI therapy, there is also evidence for an increased risk of upper gastrointestinal bleeding, which is a relevant side effect, particularly in post-stroke patients who are regularly under antithrombotic therapy [8,9]. Therefore, the potential efficacy and safety of early SSRIs therapy for PSD prevention is still conflicting.

Recently, two large RCTs were published, dealing primarily with the question of improving functional outcomes by using early SSRI therapy in patients after ischemic or hemorrhagic stroke [10,11]. Both studies examined PSD occurrence as a secondary endpoint, but data on PSD prevention have not been collated. Therefore, we aimed to perform a systematic meta-analysis including this latest evidence to analyze the efficacy and the safety profile of early SSRI therapy for preventing PSD in patients after acute stroke.

## 2. Materials and Methods

The present systematic review and meta-analysis is reported according to the Preferred Reporting Items of Systematic Reviews and Meta-Analyses (PRISMA) statement [12].

### 2.1. Eligibility Criteria

We searched for available studies fulfilling the following criteria:

- Randomized controlled trial;
- Recruitment of ischemic or hemorrhagic adult stroke patients (≥18 years old; onset <1 month), whose imaging features were in accordance with the diagnosis;
- Start of SSRI treatment within one month after stroke onset and placebo treatment as the comparison group;
- Reporting the occurrence of depression as a primary or secondary outcome;
- Reporting adverse events.

### 2.2. Outcome

Depression occurrence at follow-up was defined as the primary outcome. Secondary outcomes were adverse events that were reported by at least two of the selected studies, which included attempted suicide, bleeding event, bone fracture, acute coronary syndrome, constipation, death, diarrhea, dizziness, drowsiness, fall with injury, hyperglycemia, hypoglycemia, hyponatremia, insomnia, nausea, seizure, sexual dysfunction, and sweating.

### 2.3. Search Strategy and Study Selection

The literature search in the MEDLINE and SCOPUS databases was performed by two independent reviewers (D.R. and J.C.J.) using the following terms in combination: (stroke OR cerebrovascular accident) AND (serotonin uptake inhibitor OR citalopram OR escitalopram OR fluoxetine OR paroxetine OR sertraline OR fluvoxamine).

**Figure 1:**
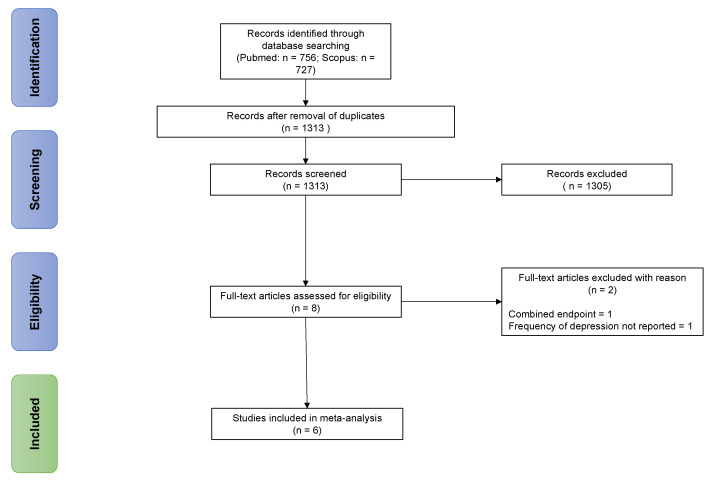
Study selection process.

Multiple reports of the same study were collated and assessed as a single study. No language or other search restriction was applied. The literature search was performed between 1 October and 19 October 2021. Reference lists of all articles that met the inclusion criteria and relevant review articles were examined to identify studies that the initial database search may have missed. Figure 1 demonstrates the figure selection process.

### 2.4. Risk of Bias Assessment

The risk of bias in included studies was assessed by the Cochrane Risk of Bias Tool for RCTs [13]. In case of discrepancies, a consensus was reached by discussion.

### 2.5. Data Synthesis and Statistical Analysis

For the statistical analysis, depression occurrence and adverse events were treated as dichotomous data. We calculated the risk ratio (RR) with the 95% confidence intervals (CIs) between the SSRIs and placebo groups. Due to the expected heterogeneity, we calculated the RR under a random-effects model (DerSimonian and Laird). We assessed heterogeneity between studies with the I^2^ statistics. Post-hoc sensitivity analysis was conducted for missing outcome data. Publication bias assessment was performed by funnel plot analysis and Egger’s test. Calculations were performed with the Stata Statistical Software Release 17 for Mac (StataCorp LP, College Station, TX, USA).

### 2.6. Subgroup Analysis

Subgroup analysis for age, stroke severity, SSRI type and dosage was planned but not conducted due to the low number of studies.

## 3. Results

A total of 1483 studies were identified, of which 6 studies with 6560 patients were finally included. The characteristics of the included studies are given in Table 1. All studies were double-blind RCTs testing SSRI (three fluoxetine, two sertraline, one escitalopram) versus placebo. The primary aim in three studies was the effect of SSRI on functional outcome, while the other three studies primary aimed to evaluate the effect of SSRI on PSD prevention. All studies used different diagnostic criteria for the detection of depression, as given in Table 1. The classification of stroke type (ischemic versus hemorrhagic) was not reported in one study [4]. The follow-up period varied between 3 months and 52 weeks. All studies reported the incidence of adverse events after randomization.

**Figure 2:**
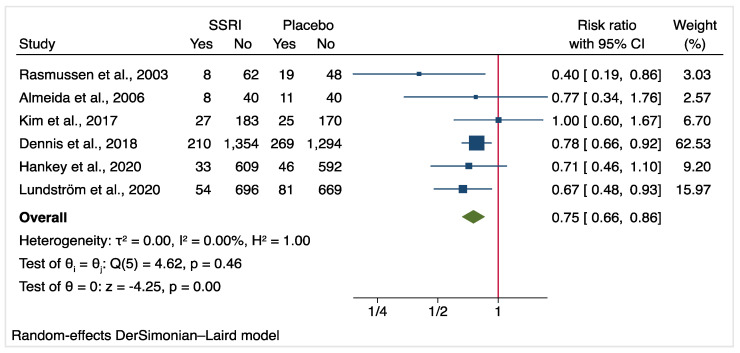
Forest plot of PSD occurrence comparing SSRI versus placebo.

The meta-analysis of the six included RCTs revealed a reduction of PSD incidence in the SSRI group compared to placebo (10.4% versus 13.8%; RR: 0.75 [95% CI, 0.66–0.86]; absolute risk reduction: 3.4%; Figure 2). There was no heterogeneity between the studies included (I^2^ = 0; *p* = 0.46). For the incidence of PSD, no evidence of publication bias was revealed (Egger’s test: *p* = 0.493, funnel plot Figure S1 in the data supplement).

**Figure 3:**
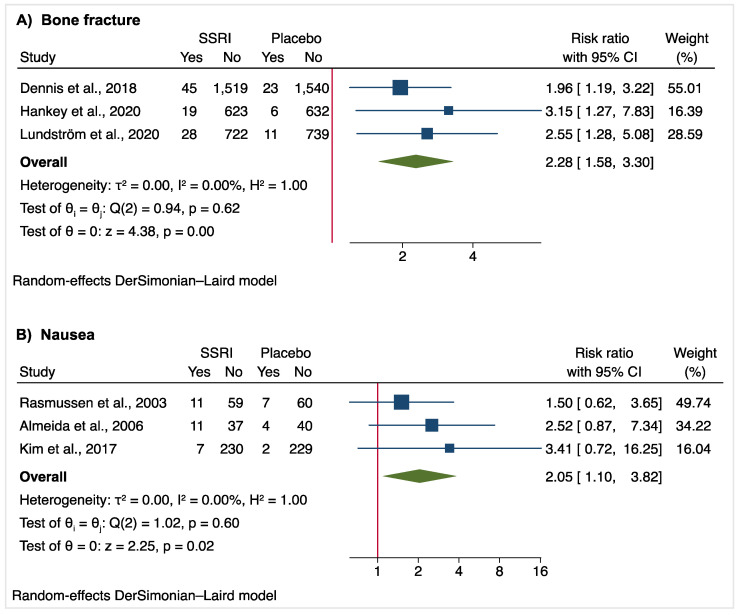
Forest plot of significant adverse events of SSRI treatment.

SSRI treatment was associated with a significant increase in the risk of bone fractures (3.1% versus 1.4%; RR: 2.28 [95% CI, 1.58–3.30]; absolute risk increase: 1.7%; Figure 3) and nausea (8.2% versus 3.8%; RR: 2.05 [95% CI, 1.10–3.82]; absolute risk increase: 4.4%; Figure 2). There was no heterogeneity between the studies for these secondary outcomes (bone fracture: I^2^ = 0, *p* = 0.62; nausea: I^2^ = 0, *p* = 0.60).

Important safety outcomes were not different between SSRI und placebo (acute coronary syndrome: RR 0.75 [95% CI, 0.26–2.18]; bleeding: RR 1.17 [95% CI, 0.81–1.70; death: RR 1.09 [95% CI, 0.70–1.69]; stroke: RR 0.93 [95% CI, 0.68–1.28]; overall: RR 1.01 [95% CI, 0.84–1.22]; Figure S2 in the data supplement]. The frequency of adverse events associated with blood level changes did not differ. Dose-depending effects on blood level changes could not be calculated, as all studies that reported this outcome parameter used the same SSRI and dosage (20 mg of fluoxetine daily, Figure S3 in the data supplement) [10,11,16]. Falls with injury were observed more frequently under SSRI therapy without reaching the level of statistical significance (RR 1.71 [95% CI, 0.80–3.64]). Other reported adverse events, including the risk of suicide (RR 0.92 [95% CI, 0.24–3.52]), did also not differ between SSRI and placebo (Figure S4 in the data supplement).

In general, all included studies were categorized as having a low risk of bias for the most categories except for missing outcome data, which were classified as high risk (one RCT) and some concerns because of the loss to follow-up or missing data for the outcome of PSD (Figure S5 in the data supplement). To address this concern, a post-hoc sensitivity analysis was conducted to test whether a difference in the incidence of PSD among patients with missing data could affect the calculated risk ratio of this meta-analysis. Despite inflation to 50% to overestimate the potential additional PSD occurrence among participants with missing data, the effect of SSRI on PSD prevention remained statistically significant (0.82 [95% CI, 0.76–0.91]; Table S1 in the data supplement).

## 4. Discussion

This meta-analysis provides the evidence that early SSRI therapy after stroke significantly reduces the incidence of PSD but to the disadvantage of more bone fractures and a higher rate of nausea compared to placebo. The absolute risk increase for bone fractures, as an important adverse event of SSRI therapy in the post-stroke population, was similar to the absolute risk reduction of PSD by SSRI therapy, influencing the benefit-to-risk consideration of this therapy in clinical practice [7]. Although the cause-and-effect link for the higher rate of bone fractures in the treatment arm is speculative, we observed a non-significant increase of falls with injury under SSRI therapy which should be investigated in future research. A combination with decreased bone density under SSRI therapy might also explain this observation [17]. Concerning the development of more effective and safer antidepressant drugs in the future, it might also be useful to investigate agents other than SSRIs for their potential to prevent PSD [18].

There are common risk factors of PSD, including stroke severity [3]. Four out of the six included studies reported the SSRI and placebo groups’ mean or median National Institutes of Health Stroke Scale (NIHSS). In general, the mean or median NIHSS was low in the studies of this meta-analysis (Table 1). A subgroup analysis of stroke severity was not rational due to the low numbers and the slight difference in the NIHSS between the included studies. Therefore, the meta-analysis cannot answer whether patients with higher NIHSS would benefit more from early SSRI therapy than less affected stroke patients. In this context, the result of a previous systematic analysis also confirmed that SSRI therapy reduced PSD incidence. Within this pooled analysis, 23 out of 248 (9.3%) patients treated with an SSRI developed PSD compared to 59 out of 242 (24.4%) patients treated with a placebo [19]. It should be noted that the rate of depression in the placebo group of the systematic analysis by Salter and colleagues was much higher than in this meta-analysis. This is most likely due to the heterogeneity of the study populations and the low severity of the strokes in our meta-analysis compared to the systematic analysis by Salter and colleagues. Future research should therefore consider a stroke severity-dependent effect of SSRI on PSD prevention.

Another risk factor that should be noted when interpreting the findings is sex distribution. Female patients are assumed to be at higher risk for PSD than male patients [20]. Apart from the study by Rasmussen and colleagues, all cohorts of the included studies were characterized by a lower frequency of females. Although male dominance in stroke studies is a known but unexplained observation, it might influence the PSD incidence of the included studies [21]. Therefore, an account should be taken when interpreting the calculated incidences of this meta-analysis.

A significant strength of this meta-analysis is its consideration of a high number of patients, and that it is based on double-blind RCTs with a low probability of confounding or other bias. Furthermore, searches for this review were systematically conducted to ensure the identification of all eligible studies.

On the other hand, there are some limitations to this meta-analysis. First of all, the included trials used different diagnostic criteria for the detection of depression. Only one study used the Diagnostic and Statistical Manual of Mental Disorders (DSM) criteria to define depression [11]. Of note, DSM classification of PSD is imprecise, defining it as a depressive disorder due to another medical condition which includes overlapping phenotypes of minor and major depressive disorder after stroke. All other included studies used different questionnaires and cut-offs to detect depression in their post-stroke population. Furthermore, PSD occurrence was only the primary outcome measurement in three of the included trials, and the follow-up period varied between three months and 52 weeks across the included studies. Hence, the validity of PSD detection might be different among the included studies, and the actual incidence of PSD might be higher or even lower due to various PSD detection tools and follow-up periods. Such bias increases random error and compromises the precision of the estimates. However, it should be noted that no substantial heterogeneity was detected in any of the analyses performed. The calculated estimates could also potentially be biased by missing data error that was up to 50% (e.g., in the study by Rasmussen and colleagues [14]). Nevertheless, post-hoc sensitivity analyses proved the reliability of PSD prevention by SSRI therapy even if the PSD incidence in the lost to follow-up population would have been high at 50%.

## 5. Conclusions

This meta-analysis confirms that early SSRI therapy reduces the incidence of PSD but increases the risk of bone fractures and nausea in the post-stroke population. Considering these side effects, future studies should identify risk factors for PSD to evaluate the benefit of SSRIs in high-risk patients for PSD and further improve the benefit-to-risk consideration of this therapy for use in clinical practice.

## Figures and Tables

**Table 1:** Characteristics of the included studies.

Author, Year	Patients (n)	Hemorrhagic Stroke (%)	Mean Age (SSRI/ Placebo)	Female (SSRI/ Placebo)	NIHSS (SSRI/ Placebo)	SSRI (Daily Dose)	Intervention	Outcome Measure	Follow-Up Period
Rasmussen et al., 2003 [14]	137	3.6	72/68	50%/49%	NA	Sertraline (50–150 mg)	Initiate within four weeks after onset, treatment duration 12 month	HAM-D6 score	52 weeks
Almeida et al., 2006 [15]	111	6.3	67.9/67.1	33%/38%	NA	Sertraline (50 mg)	Initiate within two weeks after onset, treatment duration 24 weeks	HADS-D score	24 weeks
Kim et al., 2017 [4]	405	NA	63.6/63.5	43%/35%	4.9/4.6 (mean)	Escitalopram (5–10 mg)	Initiate within 21 days after onset, treatment for 13 weeks	MADRS score	3 months
Dennis et al., 2018 [16]	3127	9.9	71.2/71.5	38%/39%	6/6 (median)	Fluoxetine (20 mg)	Initiate within 15 days after onset, treatment duration 6 months	MHI-5	6 months
Hankey et al., 2020 [10]	1280	14.5	63.5/64.6	36%/38%	6/6 (median)	Fluoxetine (20 mg)	Initiate within 2–15 days after diagnosis of acute stroke	PHQ-9 score	6 months
Lundström et al., 2020 [11]	1500	12.3	70.6/71	38%/38%	3/3 (median)	Fluoxetine (20 mg)	Initiate within 2–15 days after diagnosis of acute stroke	DSM-IV, MADRS	6 months

Abbreviations: NIHSS, National Institutes of Health Stroke Scale.

## Data Availability

The data presented in this study are available from the corresponding author upon reasonable request.

## Supplementary Materials

The following are available online at https://www.mdpi.com/article/10.3390/jcm10245912/s1. Figure S1: Funnel plot of the included studies; Figure S2: Forest plot of important safety outcomes; Figure S3: Forest plot of adverse events associated with blood level changes; Figure S4: Forest plot of other adverse events; Figure S5: Risk of bias assessment; Table S1: Sensitivity analysis for missing data bias.
