# Supplementary material for: Selective Serotonin Reuptake Inhibitors for the Prevention of Post-Stroke Depression: A Systematic Review and Meta-Analysis
Source: J Clin Med. 2021 Dec 16;10(24):5912. doi: 10.3390/jcm10245912 (PMC8704665; doi:10.3390/jcm10245912)
Supplement: Supplementary file 1 [file jcm-10-05912-s001.zip › jcm-1465971-supplementary.pdf]

Table S1: Sensitivity analysis for missing data bias

Study	Number		Number of patients with potentially missing data	Number of PSD observed	Additional PSD assuming 20% of patients with missing data had a PSD	Total estimated PSD at 20%	Calculated risk ratio and 95% CI at 20%	Additional PSD assuming 50% of patients with missing data had a PSD	Total estimated PSD at 50%	Calculated risk ratio and 95% CI at 50%
Rasmussen et al., 2003	SSRI	70	35	8	7	15	**0.783 (0.693-0.884)**	18	26	**0.820 (0.735-0.913)**
	Placebo	67	35	19	7	26		18	37	
Almeida et al., 2006	SSRI	48	NA	8	NA	NA		NA	NA	
	Placebo	51	NA	11	NA	NA		NA	NA	
Kim et al., 2017	SSRI	210	67	27	13	40		34	61	
	Placebo	195	73	25	15	40		37	62	
Dennis et al., 2018	SSRI	1564	140	210	28	238		70	280	
	Placebo	1563	140	269	28	297		70	339	
Hankey et al., 2020	SSRI	642	30	33	6	39		17	48	
	Placebo	638	17	46	3	49		23	55	
Lundström et al., 2020	SSRI	750	38	54	8	62		19	73	
	Placebo	750	29	81	6	87		15	96	

To test whether a difference in the incidence of PSD among patients with missing data could affect the calculated risk ratio of the meta-analysis, a post-hoc sensitivity analysis was conducted. If PSD were more common in patients with missing data, this could alter the calculated risk ratio towards no effect. The PSD rate was calculated with 20% and inflated to 50% to overestimate the potential additional PSD among participants with missing data. These additional PSD were added to the observed PSD in each group. Recalculation of risk-ratios remained statistically significant at both 20% (RR: 0.783, 95% CI 0.693 to 0.884) and 50% (RR: 0.783, 95% CI 0.693 to 0.884). Therefore, the missing data of the included studies did not significantly alter the findings of this meta-analysis on PSD prevention through SSRI treatment.

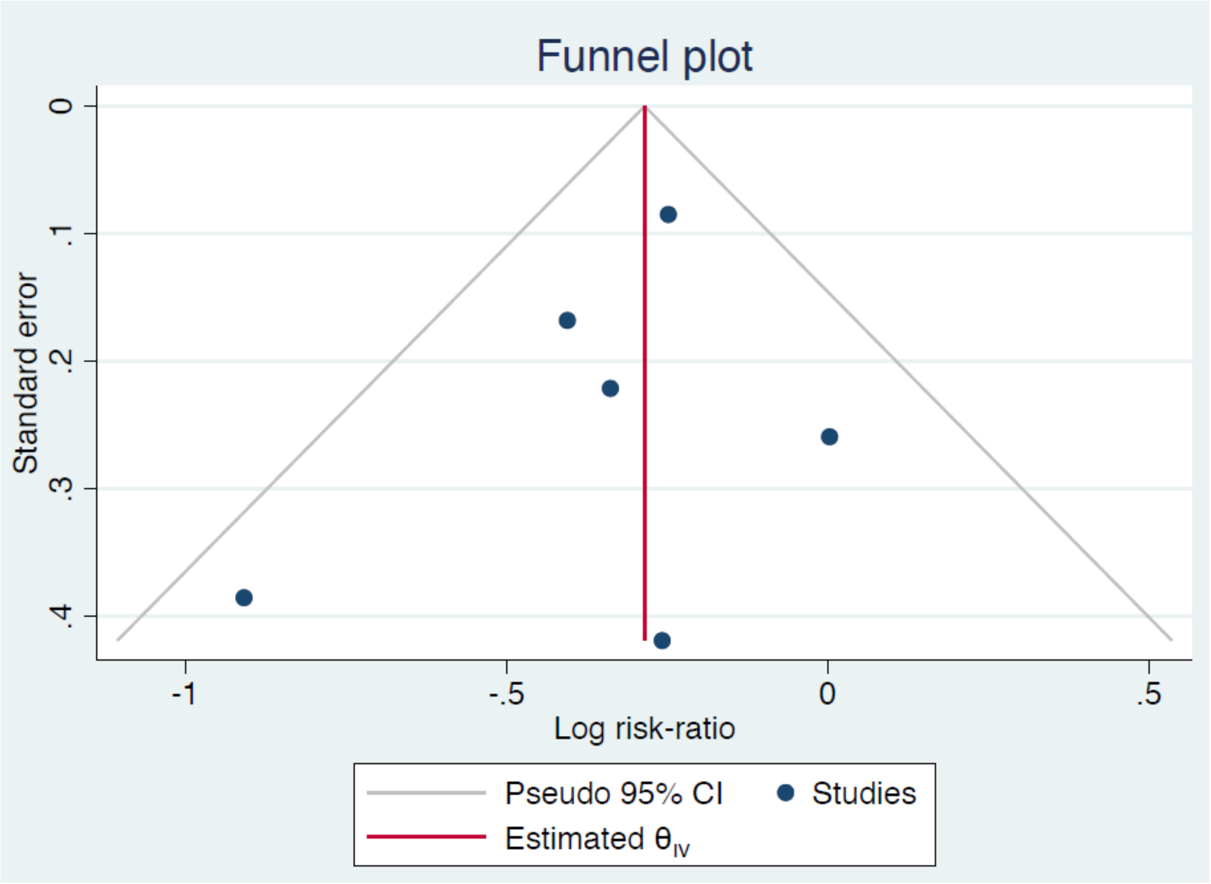

Figure S1: Funnel plot of the included studies

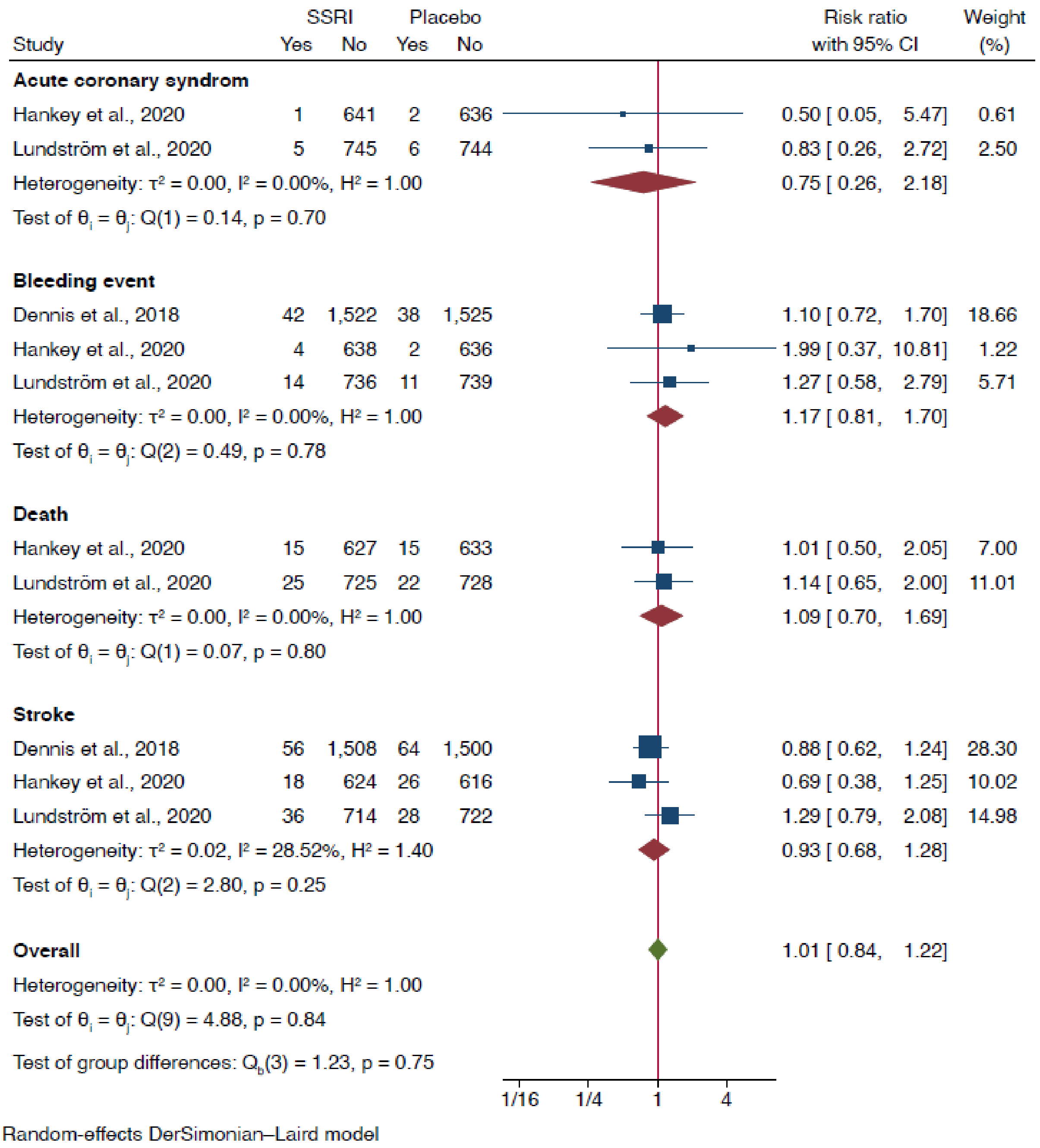

Figure S2: Forest plot of important safety outcomes

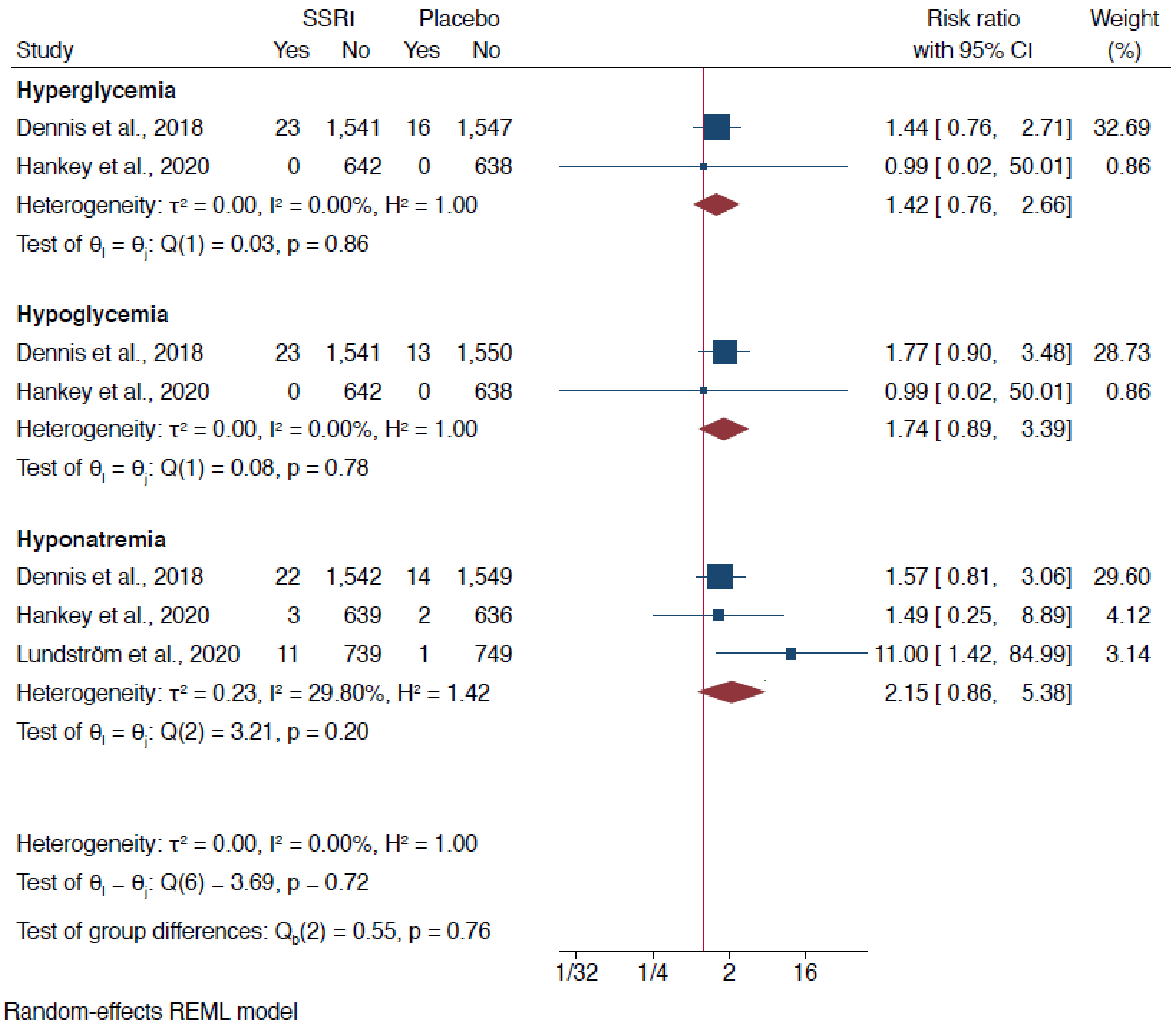

FigureS3: Forest plot of adverse events associated with blood level changes

Study	SSRI Yes	SSRI No	Placebo Yes	Placebo No	Risk ratio with 95% CI	Weight (%)
**Attemped suicide**						
Dennis et al., 2018	3	1,561	2	1,561	1.50 [0.25, 8.96]	1.04
Hankey et al., 2020	0	642	2	636	0.20 [0.01, 4.13]	0.36
Lundström et al., 2020	1	749	1	749	1.00 [0.06, 15.96]	0.44
Heterogeneity: $\tau^2 = 0.00$, $I^2 = 0.00\%$, $H^2 = 1.00$					0.92 [0.24, 3.52]	
Test of $\theta_i = \theta_j$: Q(2) = 1.27, p = 0.53						
**Constipation**						
Rasmussen et al., 2003	8	62	11	56	0.70 [0.30, 1.62]	4.32
Kim et al., 2017	14	217	14	223	1.03 [0.50, 2.10]	5.81
Heterogeneity: $\tau^2 = 0.00$, $I^2 = 0.00\%$, $H^2 = 1.00$					0.87 [0.50, 1.51]	
Test of $\theta_i = \theta_j$: Q(1) = 0.47, p = 0.49						
**Diarrhea**						
Rasmussen et al., 2003	11	59	7	60	1.50 [0.62, 3.65]	3.97
Kim et al., 2017	2	229	7	230	0.29 [0.06, 1.40]	1.35
Heterogeneity: $\tau^2 = 0.92$, $I^2 = 68.63\%$, $H^2 = 3.19$					0.76 [0.16, 3.68]	
Test of $\theta_i = \theta_j$: Q(1) = 3.19, p = 0.07						
**Dizziness**						
Rasmussen et al., 2003	16	54	16	51	0.96 [0.52, 1.76]	7.80
Almeida et al., 2006	3	45	5	39	0.55 [0.14, 2.17]	1.73
Kim et al., 2017	8	229	10	221	0.78 [0.31, 1.94]	3.77
Heterogeneity: $\tau^2 = 0.00$, $I^2 = 0.00\%$, $H^2 = 1.00$					0.85 [0.53, 1.36]	
Test of $\theta_i = \theta_j$: Q(2) = 0.57, p = 0.75						
**Drowsiness**						
Almeida et al., 2006	8	40	6	38	1.22 [0.46, 3.24]	3.32
Kim et al., 2017	1	236	1	230	0.97 [0.06, 15.49]	0.44
Heterogeneity: $\tau^2 = 0.00$, $I^2 = 0.00\%$, $H^2 = 1.00$					1.19 [0.47, 2.99]	
Test of $\theta_i = \theta_j$: Q(1) = 0.02, p = 0.88						
**Fall with injury**						
Dennis et al., 2018	120	1,444	94	1,469	1.28 [0.98, 1.66]	25.39
Hankey et al., 2020	20	622	7	631	2.84 [1.21, 6.67]	4.25
Heterogeneity: $\tau^2 = 0.22$, $I^2 = 67.59\%$, $H^2 = 3.09$					1.71 [0.80, 3.64]	
Test of $\theta_i = \theta_j$: Q(1) = 3.09, p = 0.08						

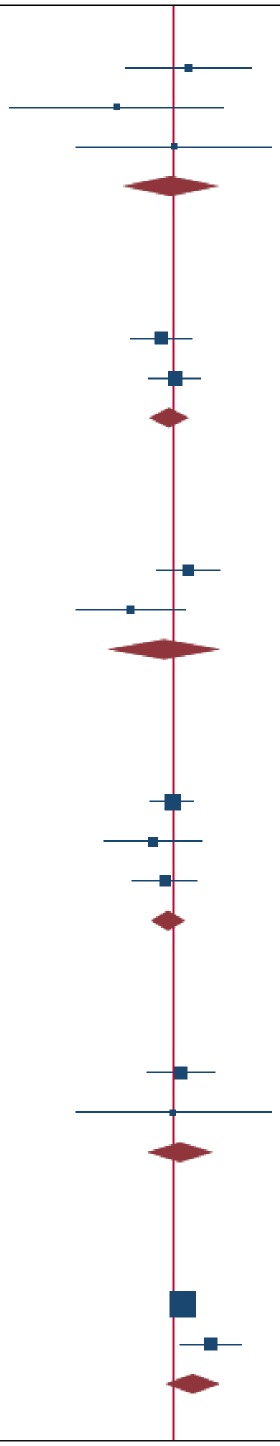

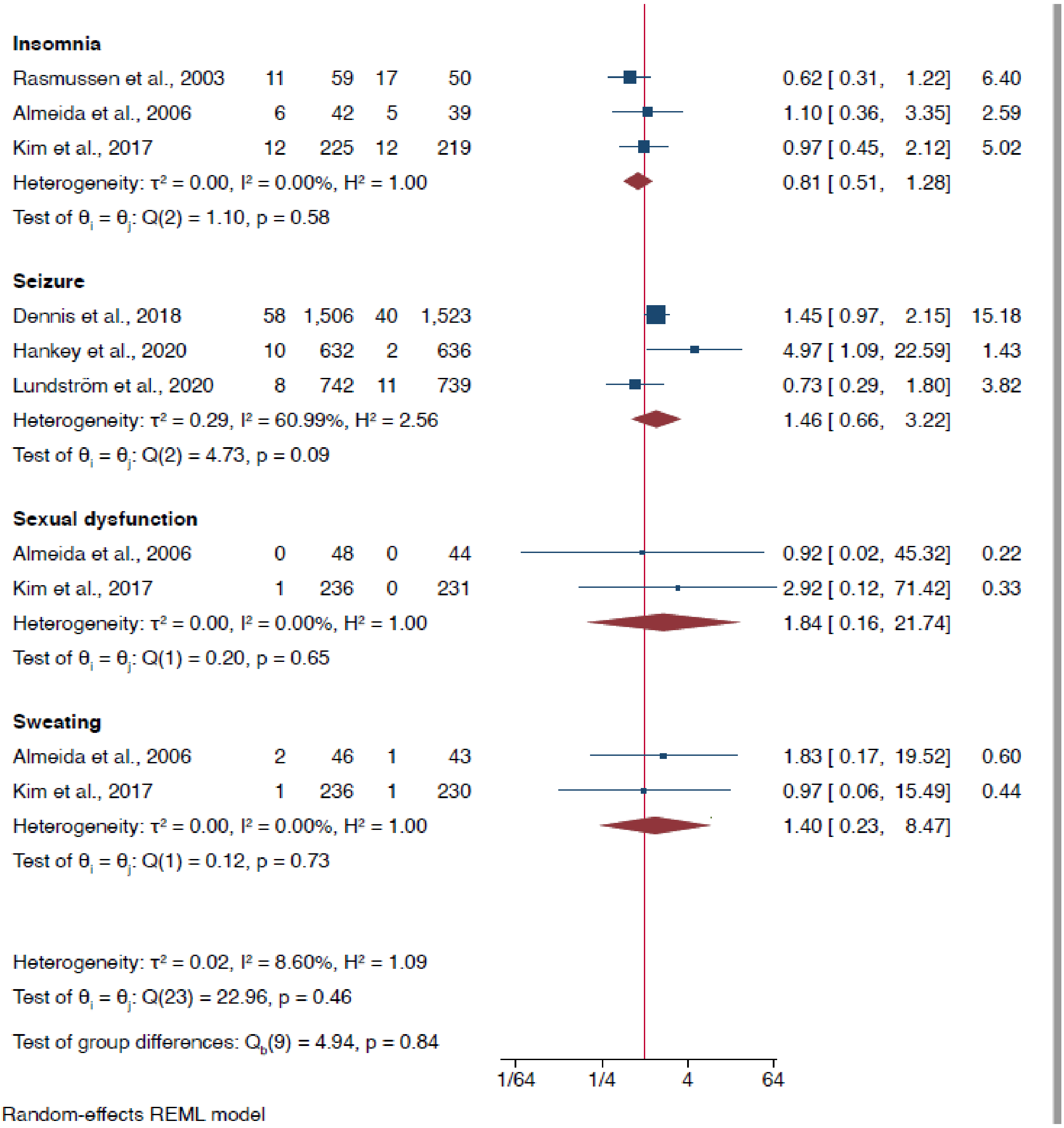

FIGURE S4: Forest plot of other adverse events

Study	Randomization process	Deviations from intended intervention	Missing outcome data	Measurement of the outcome	Selection of the reported result	Overall bias
Rasmussen et al., 2003	Unclear	Unclear	High	Low	Low	Some concerns
Almeida et al., 2006	Low	Low	Unclear	Low	Low	Some concerns
Kim et al., 2017	Low	Low	Some concerns	Low	Low	Some concerns
Dennis et al., 2018	Low	Low	Some concerns	Low	Low	Some concerns
Hankey et al., 2020	Low	Low	Some concerns	Low	Low	Some concerns
Lundström et al., 2020	Low	Low	Some concerns	Low	Low	Some concerns

FIGURE S5: Risk of bias assessment
